# The Contribution of Sustainable Tourism to Economic Growth and Employment in Pakistan

**DOI:** 10.3390/ijerph16193785

**Published:** 2019-10-08

**Authors:** Faiza Manzoor, Longbao Wei, Muhammad Asif, Muhammad Zia ul Haq, Hafiz ur Rehman

**Affiliations:** 1 Department of Agricultural Economics and Management, School of Management, Zhejiang University, Hangzhou 310029, China; faiza1885@yahoo.com or; 2 School of Public Affairs, Zijingang Campus, Zhejiang University, Hangzhou 310058, China; asif.ma015@gmail.com or; 3 School of Management, Zhejiang University, Hangzhou 310029, China; ziasm111@yahoo.com; 4 Department of Economics, Hazara University, Mansehra 21120, Pakistan; hafizkhattak@gmail.com

**Keywords:** sustainable tourism, economic growth, employment, gross domestic product, Pakistan

## Abstract

In the global economy, tourism is one of the most noticeable and growing sectors. This sector plays an important role in boosting a nation’s economy. An increase in tourism flow can bring positive economic outcomes to the nations, especially in gross domestic product (GDP) and employment opportunities. In South Asian countries, the tourism industry is an engine of economic development and GDP growth. This study investigates the impact of tourism on Pakistan’s economic growth and employment. The period under study was from 1990 to 2015. To check whether the variables under study were stationary, augmented Dickey–Fuller and Phillips–Perron unit root tests were applied. A regression technique and Johansen cointegration approach were employed for the analysis of data. The key finding of this study shows that there is a positive and significant impact of tourism on Pakistan’s economic growth as well as employment sector and there is also a long-run relationship among the variables under study. This study suggests that legislators should focus on the policies with special emphasis on the promotion of tourism due to its great potential throughout the country. Policy implications of this recent study and future research suggestions are also mentioned.

## 1. Introduction

The tourism industry has emerged as a key force for sustainable socioeconomic development globally [1,2]. The idea behind sustainable tourism is to visit the locations without harming the local community and nature and also having some constructive impact on the environment, society, as well as the economy of the country [3]. Tourism can include transportation to the general place, local transportation, accommodations, leisure, entertainment, shopping, and nourishment. It can be linked to travel for recreation, business, family, and/or friends [4,5]. Currently, there is a widespread consensus that tourism growth should be sustainable, although the question of how to achieve this is a subject of debate [6].

Tourism and the travel sector are important economic activities all over the world [7]. In many countries, the tourism industry remains an important source for the generation of employment and income in formal and informal sectors [8]. For instance, Hwang and Lee [9] claimed that economic growth and development is rapidly increasing in Korea due to the increase in elderly tourism. This increase shows that tourists feel inner satisfaction, which positively affects their future behavioral intentions [10]. Similarly, developing countries can engender a huge amount of foreign exchange from tourism that could also boost their sustainable growth and development [11]. In developing countries, it is the main source and a foundation for a country’s economic development and growth [12]. Tourism revenue complements the exchange derived from the overseas trade of goods and services. This sector also finances capital good imports in the development of the economy’s industrial sector. Alternatively, economic expansion in the developed nations influences business travel (overseas visits), which can lead to a rise in the nation’s overseas reserves [13].

International tourism has become increasingly important in several nations around the globe [14]. As per the report of the WTO (World Tourism Organization) in 2018, international tourists spent $1.3 billion per day and in total $462 billion in the year 2001 only. In most of the countries, the revenue from tourism is considered as a substitute for export earnings and contributes a lot to their balance of payment [15]. The government can generate revenue and also enhance household income through development of this sector and easing austere visa policies for international visitors/tourists. There are a lot of examples where tourism has a very positive impact on the economy of any country [16].

In the globalization era, third world nations started tourism to advance their economy, promote peace, develop human resources, and reduce the poverty level [17]. Tourism helps to “enhance employment opportunities and earnings, which can be of major economic significance to the local population” [18]. In terms of employment, the local community could expand their earnings and socio-economic condition, which could lead to an improved standard of living. Tourism improves local community development and helps to reduce poverty [19].

### Tourism in South Asia

South Asia is recognized as a distinctive region with a substantial contiguous landmass, and diverse physical features from grasslands to forests, and swamplands to deserts. It has a large variety of natural resources, coastal areas, mountains, scenic beauty, and rivers, and assorted climatic conditions, which makes this region even more attractive [20]. In South Asia, there are eight counties, namely: Pakistan, Bangladesh, Sri Lanka, Nepal, Maldives, India, Afghanistan, and Bhutan. In these countries, the economic base is still weak despite such great potential for tourism. The tourism industry is also a tool of economic development in South Asia like other developing countries.

**Figure 1:**
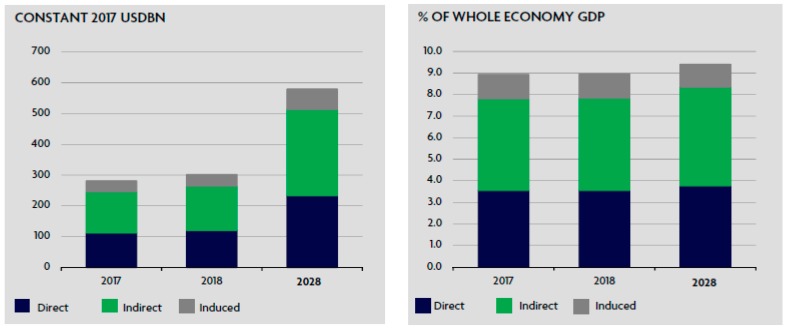
Source: World travel and tourism council, 2018. SOUTH ASIA: Total contribution of travel and tourism to GDP.

**Figure 2:**
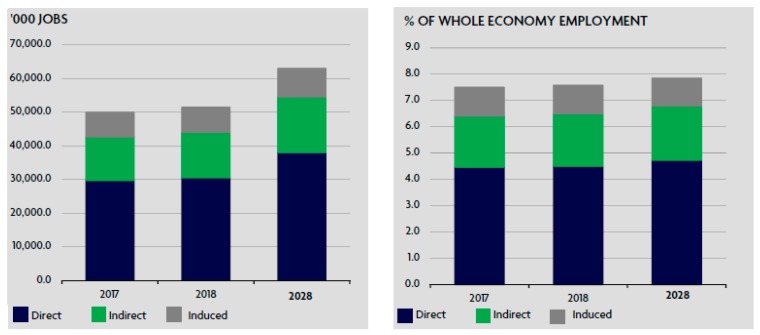
Source: World travel and tourism council, 2018. SOUTH ASIA: Total contribution of travel and tourism to employment.

In the South Asian region, the share of the tourism sector in GDP was 8.9% ($281.6 billion) in 2017 with the speculation of further growth of 7.2% in 2018. It will be 9.0% of GDP ($301.8 billion) with auxiliary income of 6.2% by the year 2028 to reach 9.4% of GDP ($579.6 billion), as presented in Figure 1. In 2017, tourism contributed 7.5% of jobs to the employment sector (49,931,500 jobs) and a speculated increase of 3.0% or a total share of 7.6% of the job market in 2018 (51,436,500 jobs). By the year 2028, the share of tourism in the job market is expected to touch 7.8% of the job market (63,006,000 jobs), as presented in Figure 2 [5].

In the South Asia region, Pakistan is well known for its tourism. Pakistan is a very beautiful country, and the tourism industry is growing [21]. Pakistan offers much allure in the developing world. The cultural and historical inheritance is very evident in this ancient region. The country receives much tourist attraction at Jaba, Kalam, Swat, Balakot, Malam, Shangla, Murri, Ayubia, Gilgit, Chitral, Paras, Sharan, Shinu, Lulusar, Dudupatsar, Naran, Shogran, and Kaghan valleys, Lake Saif ul muluk, Malika Parbat (highest mountain of Kaghan valley and also called Queen of the Mountains), Supat valley, and other historical mountain ranges in the country [22]. In the Northern Area of Pakistan, there are a lot of places that are comprised of majesty and grandeur. These areas have relics of distinct lands exclusive to its heritage and it is hallowed as a top destination with an astonishing collection of many attractive rivers, mountains, lakes, and valleys [23]. The Karakoram, Hindukush, Himalaya, and Pamir mountain ranges create formidable areas in the northern regions. They bring trekkers, climbers, hikers, and mountaineers. They also contain numerous unheeded rocks and flowing streams that bring hundreds of thousands of tourists every year. Only a few regions in the world can present a high-class combination of magnificent natural attraction, a rich variety of socio–economic systems, and history as offered by Himalayan and Hindukush regions of Pakistan [20].

International tourism in Pakistan has achieved record growth. The number of tourists has reached 808,000 from all tourist producing market places. This figure is a 24.4% rise from the previous two years. According to the report, in 2017, 1.75 million visitors traveled to Pakistan. The Pakistan Tourism Development Corporation (PTDC) confirmed that 30% of tourists were national (domestic) and almost 90% of the tourists preferred to journey by road; only 8.5% and 1.8% traveled by train and air, respectively. In 2017, the WTTC (World Travel and Tourism Council) mentioned that Pakistan’s tourism revenue was 19.4 billion US dollars and made up 6.9% of the GDP. The WTTC expects that amount to rise to 36.1 billion dollars by 2030. In 2016, tourism contributed 6.0% to total employment and in 2017 it increased to 6.3%. This total is expected to rise in 2018. The success of tourism in Pakistan hopes to contribute to the reduction of its poverty level. Tourism has an encouraging influence on Pakistan’s economic growth and still continues to grow [24,25].

The current study observed how the tourism industry supported the economy and also increased employment in South Asia. In South Asia, there are eight countries, but we have chosen Pakistan only for this study. In other countries, many studies have been conducted by other scholars [26,27,28,29]. However, in South Asia, less research attention has been paid towards the tourism sector and the growth of the economy. Therefore, the present study examines the relationship of sustainable tourism to economic growth and employment in Pakistan. The data was available for Pakistan but missing for other Asian countries.

This article contains six sections. The first section is the introduction of the study. The second section contains theories and hypotheses development. The third section provided the research methodology of the study. The fourth is the results section. The fifth section comprises the conclusions and discussions. The final section includes policy implications, limitations, and future research directions.

## 2. Development Theories 

Since the 1960s, the tourism sector has been considered an effective developmental growth pole, and many countries have enhanced their tourism sector to improve their economic development [30].

Todaro and Smith (2011) proposed in their study that it is a multidimensional process as far as development is concerned, and it comprises positive changes not only in economic growth and national institutions but also in poverty reduction. Modernization theory (MT) is one of the most popular developmental paradigms to have gained admiration from the late 19th till the mid-20th century. This theory is thought to be an extension of another theory called growth theory, which is grounded in Keynesian economics [31]. For justification of MT, the theorists used it as a key social indicator for economic development, which trickles down to the grassroots level of society in the form of plentiful economic and employment prospects. Wealthy and powerful modernized economies usually provide a high-quality of life and modern technology to their citizens. Modernization becomes more favored due to its bold and effectual production methods. Moreover, from the tourism perspective, the modernization strategies of development not only engender foreign capital but also smooth the way for the transfer of technology and create greater employment opportunities than before. The main focus of tourism development is these economic paybacks, and whenever other economic resources trickle down, the tourism multiplier acted as a growth-pole [26].

Dependency theory (DT) became popular in the 1960s and 1970s. It is a composite of numerous interrelated theories and mainly focuses on the inequalities of core capitalist and southern developing countries [32]. According to this theory, historically poor countries are kept deprived of development by developed and rich countries. The economic reliance of developing countries on development projects is based on: (1) transfer of advanced technology from industrialized countries; (2) creation of massive debts and dependency on foreign investment; and (3) interest on debts transmitted back to the developed countries [33].

In response to the huge criticism of this theory in the 1970s and 1980s, neoliberalism theory (NL) was formed. NL is a theory of free global markets without any economic and political influence. The dogma behind this theory is to remove all barriers constructed by developed countries. There should be a free flow of capital and trade globally and slight consideration should be given to the market traits like privatization, market development, de-regularization, self-determination, and self-sufficiency [34]. In the above-mentioned era, it was widespread that governments had the capabilities to earn foreign capital. As far as neoliberal tourism is concerned, hotel chains could play a primary role to magnetize foreign capital. By developing infrastructure in tourist attraction areas, the local and national economies could get a boost, although the decrease in state participation in social welfare programs and limiting trade unions, the spending on education, health, environment, and other welfare also decreased. In Neoliberal, tourism not only pauses the development of the state but also humiliates and exploits the labor to set off its own self-interest. Due to the many consequences, state representatives hesitate to start projects of human development as well as programs for the local community’s well-being [30].

Eventually, in the decades of the 1970s and 1980s, it was realized that rather than benefiting the poorest of the world, policies were tilted, i.e., global top-down approaches were in favor of the west only. To enrich and uplift the standard of living of the poorest, aid and development organizations started searching for causes of poverty [35]. Instead of getting rid of the root causes of poverty, the new alternative development (AD) or bottom-up style was launched only to minimize the symptoms. A basic needs approach (BNA) only concentrated on basic needs and did not focus on economic development.

According to neoliberal development and general modernization theories, the standard of living is measured by economic growth. However, DT and AD theories of economic development interrogated this approach. The DT approach demonstrated the negative effects of Western development on the poorest communities of the south and AD theory swung development to bottom-up from the top-down approach.

Sustainable development (SD) is the combination of two different objectives of sustainability and development. In simple words, this theory is a mixture of development and sustainability theories [36]. Until the end of the 20th century, ecologists were concentrating on the ideas of the conservation movement of the 19th century [37]. Tourists with environmental knowledge loathe ecological and social damage. The sustainability factor has been appropriated irrespective of the fact that the tour operating companies were motivated by promoting ecological vacations. Ioannides [38] was of the opinion that the International Federation of Tour Operators (IFTO) used sustainability as a marketing tool with the intention to maximize profit. Currently, efforts are being made for a reduction in poverty through pro-poor tourism. In attaining the UN Millennium Development Goals, the UN WTO (2005) report blatantly supports the role of community tourism through the local private sector.

### Empirical Literature Review and Hypotheses

Researchers have a variety of views concerning how tourism contributes to economic expansion and employment in many developed and underdeveloped countries. A number of scholars have the same opinion on the significant role of tourism and how it relates to the growth of the economy. According to the World Tourism Organization, tourism is an activity of traveling for the purpose of leisure outside the day to day environs. The benefits received by local and national economies should be analyzed accordingly [39]. For economic development, tourism has been considered one of the driving forces. It has a positive impact in creating a foreign exchange and generating employment opportunities and local revenue [40,41,42]. Numerous studies in different less-developed nations around the world have found a significant correlation between tourism and economic expansion [41,43,44].

Ayeni et al. [39] explored the growth of sustainable tourism in Nigeria. According to the authors, tourism has become an instrument for diversifying the economy for several countries. This has supported the service sector and has created a major connection with Nigeria’s economy, by promoting new employment opportunities and creating new sources for revenue generation. However, the developed nations have a higher ratio of world tourism in comparison to less-developed nations; but still, there are a lot of opportunities for less developed countries to get maximum benefits from this industry. The researchers analyzed through the qualitative technique of research the potential of Nigeria’s tourism on its economy and found that the industry has great potential but is yet to be explored. They were of the opinion that given the endeavors of the government to eradicate poverty and diversify the economy, tourism could contribute a lot.

Manwa [45] posited that in order for tourism to be sustainable for society they must gain economically from it. This would allow them to protect and maintain the popular tourist areas. This is also highlighted by Smith [46]: that the economic benefits of tourism depend on the country’s aptitude to offer appropriate and adequate amenities.

Brida [47] emphasized the impact that tourism had on economic expansion in Chile. The purpose was to examine a probable causal association between exchange rate, tourism expenditure, and economic expansion for the period 1986 to 2007. The hypotheses were empirically analyzed by employing the Johansen Cointegration Test. The author found that tourism and economic expansion had a positive correlation and that tourism was the main contributing factor to economic expansion. Pavlic et al. [48] revealed the impact of tourism on employment in Croatia. According to the authors, the tourism sector contributed to the promotion of employment after examining quarterly data from 2000−2012 through Johansen Co-integration Test and Granger Causality Test. The researchers also found a positive impact of tourism on employment and co-integration showed a long-term correlation amongst the variable.

Wang et al. [49] investigated the correlation between GDP and tourism revenues in Guizhou, China. Findings of their study showed a significant and positive correlation between tourism and GDP. Akan et al. [50] demonstrated causal relationships among the tourism sector and economic development of Turkey. The researchers used the Granger Causality test, Phillips–Perron test, the co-integration approach, and a Vector Autoregression (VAR) model for the time period 1985–2007. Findings of the test revealed that the tourism industry in Turkey was positively affected by economic development. The analysis indicated that a long-term steady correlation among economic development and tourism growth exists.

Kreishan [51] analyzed the causality association among tourism revenues and economic development (GDP) for Jordan. The author covered the annual time series data of 1970–2009 for analysis. To check the causal association, the researcher used the Augmented Dickey–Fuller (ADF), Johansen Co-Integration, and Granger Causality tests. The results showed that there was a positive relationship in the long term among economic expansion and tourism growth. The existence of a direct connection among tourism revenue and economic growth was also observed through the Granger Causality test.

Adnan et al. [23] estimated the long-run relationship amongst tourism revenue and economic development in Pakistan. The authors used the annual data from 1971 to 2008 for analyses. The findings confirmed the long-term relationship among revenue from tourism and economic development, and in their study, they explained that revenue from tourism led to increasing economic growth in Pakistan, except between 2006 and 2008.

Sr et al. [52] explained the connection among eradication of poverty, tourism growth, and economic development in Nicaragua. The researchers found a direct relationship between tourism growth and poverty eradication. The authors characterized the association amongst the variables under study as they related to “democratization of the dollar”. They highlighted the employment and income opportunities that are derived from a transfer of income and wealth from the inhabitants of developed countries compared to less-developed countries. According to the Shan et al. [53] and Kulendran et al. [43] in their analyses of China and Australia, they observed that there is a strong association between international travel and trade. A Korean case suggests that economic development can attract many business tourists. The case also suggests that economic growth can lead to tourism expansion.

Several studies have shown the direct connection between international trade (particularly export expansion) and economic growth [54,55,56]. The authors have seen a robust relationship between international trade and economic development and also a strong correlation between exports and economic growth. Moreover, tourism extensions are connected to economic development. However, export-oriented economic development caused tourism income to drop. Lastly, the strategies of continuous promotion of tourism may not be as effective as perceived by decision makers, if no direct relation was found between tourism development and economic growth, because it generally happens when tourism development has a positive impact on the economy [45]. In the South Asian region, especially in Pakistan, studies of tourism’s effects on economic growth have been less evident in tourism related literature. Therefore, we examined the relationship between growth of tourism, economic development, and employment in the context of Pakistan. Hence, our research is based on the following hypotheses:

**Hypothesis 1** **(H1).** *There is a positive association between annual tourism growth and GDP.*

**Hypothesis 2** **(H2).** *There is a positive association between annual tourism growth and an increase in the employment rate.*

## 3. Research Methods

### Data and Variables

To study the contribution of sustainable tourism to economic growth and employment in Pakistan, the annual time series data from 1990 to 2015 was taken for analysis. In this study, the annual growth of tourism was taken as an independent variable and both employment and GDP were used as dependent variables. Due to the time series data, this study may exhibit some stationary or non-stationary variables. In this study before determining that all the series were integrated, a unit root test (i.e., ADF) was applied.

Firstly, the bivariate regression model was used for quantitative analysis to investigate the empirical relationship between two variables and hypothesis testing [57]. Secondly, to find a long-run relationship between variables, we used co-integration analysis. For statistical analysis and also for econometrics model estimation, E-view 9 was used. For data collection, different sources were used, i.e., Tourism Year Book, Economic Survey of Pakistan, Ministry of Tourism Government of Pakistan, World Travel and Tourism Council, and Tourism Economic Impact annual reports.

## 4. Results

### 4.1. Descriptive Statistics and Correlation Matrix

The variables’ descriptive statistics and correlation matrix are presented in Table 1. Means, standard deviation, and correlations are revealed in the table. These results of the correlation matrix aligned with the previous study [58,59]. Generally, multicollinearity was very low and did not present a serious concern [60].

### 4.2. Unit Root Test Results

Testing for a unit root of the variables was necessary in order to rule out the possibility of non-stationarity of the data. Therefore, the commonly accepted ADF and Dickey and Fuller unit root test was adopted to check the stationarity of the GDP, employment, and annual tourism growth (Tour_g) series. The test was based on an estimate of the following regression:

$$\Delta y_{t}=\dot{a}+\delta y_{t-1}+\overset{p}{\underset{\dot{i}=1}{\sum }}\beta \Delta y_{t-1}+e_{t}$$

where Δ is the first difference, Y is the time series, *t* symbolizes linear time trend, α denote a constant, *n* is the number of lags on predicted variables, and *e* represents the error term.

In Table 2, the results show that the level value of all three sequences was non-stationary and further tested signposted that GDP, EMP, and Tour_g were stationary at the first-order difference. The first-order difference was made on three sequences to reduce fluctuations of the data. Then three new series, ∆GDP, ∆EMP, and ∆Tour_g, were obtained and their unit root test results are shown in Table 3.

In Table 2 and Table 3 the results of ADF and PP both indicated that GDP, employment, and annual tourism growth were not stationary in their level form but stationary at first level. Thus, both test variables were integrated of the same order1 (1).

### 4.3. Regression Analysis Technique

To examine the contribution of tourism to economic growth and employment, a regression analysis technique was used and the same technique was employed as in [61].

The model can be expressed as:

Y_i_ = β_0_ + β_i_ x_i_ + ε (2)

where Y_i_ = dependent variable, X_i_ = independent variable, β_0_ = intercept, β_i_ = coefficient to be estimated.

The proposed modified regression model is represented by the following equation:

#### Model Specification

ΔGDP = β_0_ + β_1_ΔTou_g + ε (3)

ΔEmp = β_0_ + β_1_ΔTou_g + ε (4)

where Δ = first difference, GDP = gross domestic product, Emp = employment rate, Tou_g = annual tourism growth, β_0_ = intercept, β_S_ = coefficient to be estimated, and ε = error term.

From Table 4 the results show that the *p*-value was 0.000, which is less than 0.05 (*p* < 0.05), which indicates that there was a significant contribution to the annual growth of tourism to the GDP. Moreover, the values of *t*-statistics were also above the cutoff value of 1.96 [62,63]. The R-squared value meant that there was a 5% variation in GDP due to annual tourism growth. There was also a significant and positive relationship between explanatory variables and the predicted variables because the *T*-values were greater than 1.96. These results also aligned with the study of Kim et al. [56].

Table 5 shows that the dependent variable was employment rate and annual tourism growth was the independent variable. According to the analysis, the *p*-value (0.04) was less than 0.05. The findings demonstrate that annual tourism growth contributed significantly to the employment rate. The R^2^ value implies that 15 percent variation in employment rate was due to tourism growth. The beta coefficient was positive, which entailed that annual tourism growth and employment rate had a significant and positive relationship; consequently, we accepted the alternative hypothesis and rejected the null. Richardson [64] confirmed the same patterns results in his study.

### 4.4. Findings of Cointegration Test

To investigate the stable long-run relationship between annual GDP, employment, and annual growth of tourism, Johansen’s Cointegration test was selected amongst several other techniques available for time series data. The main objective of the variables under study was to estimate the stationary linear combination.

Johansen and Johansen and Juselius used maximum eigenvalue and trace statistics to test whether there was a long-term relationship between the variables. There are numerous methods for the determination of the lagging length and the most commonly used is the Schwarz criterion. This is because Schwarz criterion has been scientifically proven [65], and the critical values are more unbiased relative to other criteria. Therefore, this study determined the lagging length based on Schwarz critical values.

For the Johansen test of cointegration, the precondition was that variables must be non-stationary at level, but our three variables were integrated of the same order.

Table 6 shows that the result of the trace test indicated a solid cointegrating relationship between the variables. The value was less than 5% so we could not reject the null hypothesis, which meant that there was cointegration amongst variables; these variables had long-run associations and in the long run, they moved together. Furthermore, the maximum eigenvalue test demonstrated all the variables co-integrated and in the long run they had an association.

Both test trace and max-eigenvalues indicated the same thing: that variables (GDP, EMP, Tour_g) were co-integrated. They had a long-run association, and for the long run, they could move together.

## 5. Discussion

Tourism is one of the fastest growing industries and also a driving force for so many developed as well as developing economies. It is the largest source of employment opportunities and a huge wealth originator and a greater contributor to the diversified economy. Weaker regions or regions in decay could be developed through the tourism sector easily. For the tantalizing economies of the South Asian countries, tourism is professed as a dynamic tool to get rid of the scarcity of development resources, such as finance and expertise.

The goal of the current study is to examine the relationship between tourism, employment, and economic development in Pakistan. We explored the positive connection between the annual tourism growth and employment and economic growth. Tourism growth can improve the employment rate as well as GDP. Kim et al. [66] demonstrated causal associations among tourism growth and economic expansion in Taiwan. The findings of their study showed a long-term equilibrium association. Sanchez et al. [67] revealed that tourism expenses primarily had caused the economic deficit, but that a positive and significant economic impact on economic expansion was found. The empirical studies highlighted the impact of the tourism demand on employment pointed out that tourism had a significant effect on employment rate [68,69]. According to Archer [70] and Mathieson [71], tourism creates direct and indirect employment opportunities. However, studies in this context are inadequate in Pakistan. As a result, we investigated this gap and found a positive impact that tourism had on employment and economic development. Findings of our study showed that tourism growth has a positive correlation to employment and GDP. Our results are compatible with the previous research findings of Khalil and Pavlic [48,72]. Moreover, the main outcomes of the present study are in line with previous research outcomes [67,70]. The result of the cointegration analysis suggests the existence of a relationship between annual tourism growth and GDP, a finding that aligns with the prior study of Pedak [73]. Additionally, the results of the cointegration analysis show the long-run relationship between the annual tourism growth and employment; these results are in line with the previous study of Dimoska [74]. These findings suggest that growth in tourism has a major role in the economic growth as well as in creating employment opportunities. Findings supported the hypotheses.

Empirical and theoretical studies have discovered that the growth of the tourism sector has a positive impact on employment. In addition, its direct effect on travel and tourism can produce additional employment opportunities through its stimulating influence in many tourism sectors. The total economic impact of tourism is healthier when the tourism sector is encouraged to acquire domestic services and goods.

## 6. Policy Implications, Limitations, and Future Research Direction

For the Pakistani economy, tourism is a motivating force. The growing tourism sector can bring much optimism to the economy, mainly in terms of income, GDP, generation of employment, and economic growth. Pakistan is a popular tourist destination. Its array of natural beauty, as well as its traditional and cultural inheritance, will play an important role in Pakistan’s future if the tourism industry develops systematically and is supported well.

The tourism sector requires creative and talented people and well-developed infrastructure in place. Policies drawn from this study are that the government should create employment opportunities, income sources, and revenue for the local inhabitants as well as economic activities in the country through the development of tourism. The government can develop the tourism industry by providing the incentive to the tourism sector in the form of basic infrastructures such as a high-quality transportation system, roads, immense airports, and tax incentives to the tourism-related industries (i.e., hotels). Political stability must be established to improve Pakistan’s image to the world. The government should also ensure the security of all tourists and formulate sustainable tourism policies. This ensures a stable, secure, and steady tourism demand for the country.

The main emphasis of the state legislators should be on a law and order situation and a quality education. Terrorist attacks not only destroy the tourism sector but also abolish the soft image of Pakistan. Globally, the country was declared as insecure for traveling. Irrespective of the poverty, unemployment, inflation, and infrastructure development, still northern areas have attracted the maximum share of tourists as compared to other areas of Pakistan. Hence, to alleviate poverty and enrich the standard of life, an international level promotion of tourism in the northern areas is needed.

There are some limitations to this study. These limitations can lead to further research. First, the recent study applied secondary data. So, future studies may focus on primary data for investigating the effect of the tourism sector on economic growth. Secondly, the present study is conducted in the context of Pakistan; future research studies should be carried out in other developing countries in terms of generalizability of the findings. Further research could also be based on a sector-driven approach in order to distinguish the direct and indirect impacts of tourism on employment. Finally, future research should encourage examining other dependent variables, specifically revenue and foreign exchange earnings, etc. Furthermore, future research is needed to additionally recognize the short-term relationship between the variables through the Johansen Cointegration and Vector Error Correction Model (VECM).

## 7. Conclusions

The main purpose of the current study is to examine the relationship between tourism to employment and economic development in Pakistan. For this study we have used time series data from the year 1990 to 2015. The annual growth of tourism was used as an explanatory variable and both employment and GDP were taken as outcome variables. Bivariate regression and Johansen cointegration technique employed for the analysis. We have investigated the positive connection between the annual tourism growth, employment and economic growth. Findings of the study showed that tourism growth plays important role in the economic development of the country.

## Figures and Tables

**Table 1:** Variable descriptive statistics and correlation matrix.

Variables	Mean	Std. Dev.	Min	Max	1	2	3
EMP	6.538	0.938	4.5	7.67	1		
GDP	9.749	3.701	2.5	13.9	0.202	1	
Tour_g	11.78	0.999	10	13.8	0.392 *	0.727 **	1

Note: EMP: Employment rate; GDP: Gross Domestic Product; Tour_g: Tourism growth; * Correlation is significant at the 0.05 level(2-tailed); ** Correlation is significant at the 0.01 level (2-tailed).

**Table 2:** Root test results of sequence level values.

Variable	ADF Level	PP Level
GDP	−2.210278	−2.450704
EMP	−4.871852	−4.922699
Tour_g	−3.740363	−3.793420

ADF: augmented Dickey–Fuller test; PP: Phillips–Perron test.

**Table 3:** Root test results of the sequence first-order difference.

Variable	ADF Level	PP Level
ΔGDP	−5.826917 (0.0004)	−5.903267 (0.0004)
ΔEMP	−10.34191 (0.000)	−14.01308 (0.000)
ΔTour_g	−9,125,041 (0.000)	−9.842097 (0.000)

Note: null hypothesis rejected at 5% significance level.

**Table 4:** Regression analysis of tourism growth and GDP.

Dependent Variable: ΔGDP				
Variable	Coefficient	Std. Error	*t*-Statistic	Prob.
C	−21.95028	6.134822	−3.577981	0.0015
ΔTour_g	2.690465	0.518887	5.185067	0.0000
R-squared	0.528348	Durbin Watson		1.330163
F-statistic	26.88492	Prob (F-statistic)		0.000026

**Table 5:** Regression analysis of tourism growth and employment.

Dependent Variable: ΔEMP				
Variable	Coefficient	Std. Error	*t*-Statistic	Prob.
C	2.206832	2.083572	1.059158	0.3001
ΔTour_g	0.367638	0.17623	2.08613	0.0478
R-squared	0.153497	Durbin Watson		0.40489
F-statistic	4.351938	Prob (F-statistic)		0.047768

**Table 6:** Johansen cointegration. Series: EMP, GDP, Tour_g Lags interval (in first differences): 1 to 4.

Unrestricted Cointegration Rank Test (Trace)				
Hypothesized		Trace	0.05	
No. of CE(s)	Eigenvalue	Statistic	Critical Value	Prob.**
None *	0.974991	94.64275	29.79707	0.0000
At most 1 *	0.456825	17.18381	15.49471	0.0276
At most 2 *	0.187754	4.366994	3.841466	0.0366
Trace test indicates 3 cointegrating eqn(s) at the 0.05 level				
* denotes rejection of the hypothesis at the 0.05 level				
** MacKinnon–Haug–Michelis (1999) *p*-values				
**Unrestricted Cointegration Rank Test (Maximum Eigenvalue)**				
Hypothesized		Max-Eigen	0.05	
No. of CE(s)	Eigenvalue	Statistic	Critical Value	Prob.**
None *	0.974991	77.45894	21.13162	0.0000
At most 1	0.456825	12.81681	14.26460	0.0836
At most 2 *	0.187754	4.366994	3.841466	0.0366
Max-eigenvalue test indicates 1 cointegrating eqn(s) at the 0.05 level				
* denotes rejection of the hypothesis at the 0.05 level				
** MacKinnon–Haug–Michelis (1999) *p*-values				

Source: Author’s calculation by using E-view 9. Eqn (equation), CE (cointegrating equation).
